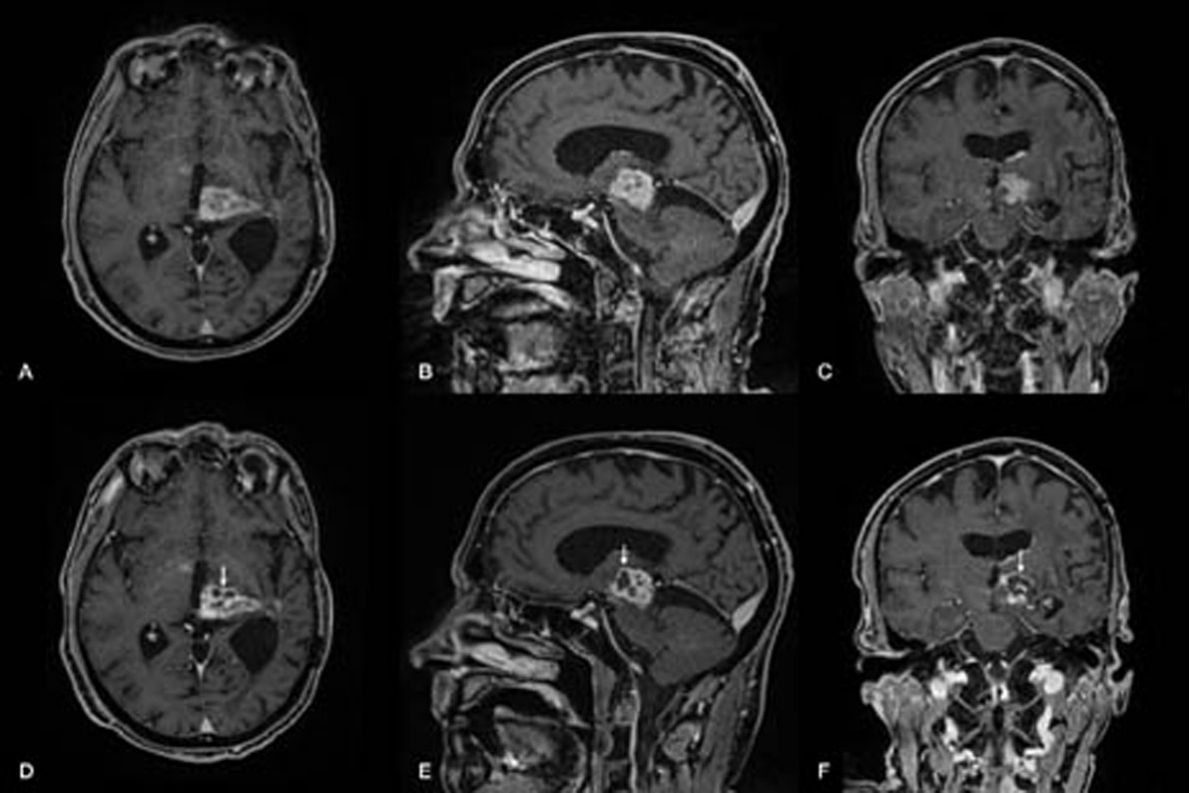# First non-invasive thermal ablation of a brain tumor with MR guided focused ultrasound

**DOI:** 10.1186/2050-5736-3-S1-O11

**Published:** 2015-06-30

**Authors:** Javier Fandino, Daniel Coluccia, Lucia Schwyzer, Javier Anon, Luca Remonda, Ruth O’Gormann, Ernst Martin, Beat Werner

**Affiliations:** 1 Kantonsspital Aarau, Aarau, Switzerland; 2 University Children’s Hospital, Zurich, Switzerland; 3 University Children’s Hospital, Zurich, Switzerland

## Background/introduction

Focused ultrasound (FUS) can penetrate soft tissue noninvasively and induce physiological effects deep within target tissues. MR-image guidance, lesioning precision, sparing of healthy tissue and absence of ionizing radiation make FUS an ideal modality for brain interventions. Based on the encouraging results in treating patients with chronic pain or movement disorders through thermal ablation of thalamic and subthalamic targets, noninvasive transcranial MR guided focused ultrasound (tcMRgFUS) recently received CE marking for functional neurosurgery. Here, we report the world’s first successful application of noninvasive tcMRgFUS for tumor ablation in a patient suffering from a centrally located malignant glioma.

## Methods

Case Report: A 63 year old patient presented in our clinic with tumor recurrence in the left thalamic and subthalamic region five years after first surgery for a glioblastoma.

After giving informed written consent he was included in our ongoing clinical phase-1 study on the feasibility and safety of tcMRgFUS for the treatment of brain tumors. Due to the location of the tumor within eloquent brain areas and considering previous radiotherapy and various cycles of different chemotherapeutics, surgical resection was excluded as a treatment option. The tcMRgFUS procedure was performed using the ExAblate Neuro^®^ system (InSightec Ltd, Haifa, Israel) integrated into a clinical 3T MR system (GE Healthcare, Little Chalfont, UK). The patient received local anesthesia for positioning a stereotactic frame (Integra LifeSciences Corporation, New Jersey, USA), and prophylactic administration of paracetamol and ondansetron for preventing pain or nausea. Post-interventional assessment included follow-up MRI immediately after and on days 1, 5 and 21 after the procedure. The patient was awake and responsive during the whole intervention with stable neurological assessment and without additional medication needed. In the 3 hours lasting procedure, a total of 25 sonications were applied with up to 19’550 Joules of acoustic energy. 17 sonications reached ablative temperatures of over 55°C with a maximum of 65°C, as recorded by intraoperative realtime MR thermometry. MRI scans acquired immediately after the intervention revealed significant lesions to the sonicated tumor tissue that were particularly well visible in DWI and ADC maps. Later on, contrast-enhanced MRI showed well-circumscribed volumes of non-perfused tissue, indicative for ablated tumor tissue. As expected from earlier experience, perifocal edema developed around the thermal lesions, but gradually disappeared during the follow-up period. Neurological examination on day 5 revealed improvement of the preexisting right arm paresis while no additional neurological deficits were observed.

## Results and conclusions

The successful ablation of a brain tumor demonstrates the feasibility of noninvasive tcMRgFUS tumor surgery. Further clinical interventions in the context of our ongoing clinical phase I study will be needed to assess the safety and efficacy of tcMRgFUS for brain tumor treatment and its relevance for future treatment strategies against brain tumors.

**Figure 1:**